# The fluorescent probe DISBAC_2_(3) provides a high-throughput screening tool for evaluating abiotic stress tolerance in plants

**DOI:** 10.1093/plphys/kiaf560

**Published:** 2025-11-04

**Authors:** Caroline Ivsic, Ping Yun, Frances C Sussmilch, Sergey Shabala

**Affiliations:** School of Biological Sciences, University of Western Australia, Crawley, WA 6009, Australia; School of Biological Sciences and ARC Training Centre for Smart and Sustainable Horticulture, University of Western Australia, Crawley, WA 6009, Australia; School of Natural Sciences, University of Tasmania, Hobart, TAS 7001, Australia; School of Biological Sciences and ARC Training Centre for Smart and Sustainable Horticulture, University of Western Australia, Crawley, WA 6009, Australia; International Research Centre for Environmental Membrane Biology, Foshan University, Foshan 528000, China

## Abstract

The electric gradient across cellular membranes (termed membrane potential [MP]) provides a driving force for the uptake and translocation of all essential nutrients in their ionic form, as well as operating in stress sensing. MP is causally associated with abiotic stress tolerance in plants and, thus, could be used as a proxy in phenotyping programs. However, the conventional method to detect MP changes, which involves impaling the membrane with a microelectrode, requires specialized equipment and specialist skills, is highly time consuming, and is prone to various possible artifacts. Here, we present a high-throughput screening approach that enables the rapid detection of MP changes in plants using the fluorescent probe DISBAC_2_(3). Using 3 case studies (salinity stress and hypoxia stress in roots and light fluctuations in guard cell movements), we benchmarked this method against conventional microelectrode impalements and demonstrated the feasibility of using this dye for a high-throughput MP screening in various cell types of different species in response to different abiotic stimuli. Through these studies, we show that a voltage-sensitive probe can rapidly and effectively measure the changes in MP elicited by fluctuating environments. We emphasize that the use of such techniques in breeding programs (rather than the time-consuming conventional methods) provides a solution to rapidly identify stress-resistant crops.

## Teaser

Rapidly screening for changes in membrane potential and for stress-tolerant crops is uncovered using the voltage-sensitive dye DISBAC_2_(3).

## Introduction

Development of molecular approaches (e.g. single-cell RNAseq or spatial transcriptomics), strengthened our understanding of how genes of interest operate in tissue- and cell-specific contexts (Chen et al. 2024; Singh et al. 2024), shedding light on molecular mechanisms related to plant development and adaptive responses to hostile environments. However, phenotyping methods for abiotic stress tolerance still rely on a whole plant assessment, making it hard to link genotype and phenotype data and account for cell specificity.

Central to cell operation is an electric gradient across cellular membranes that provide a driving force for uptake and translocation of all nutrients. At the plasmalemma, membrane potential (MP) represents the electric potential difference between apoplast and symplast that is maintained by the different interior and exterior distributions of ions in a cell. The resting MP is always negative and is largely conferred by the activity of the proton pump (H^+^-ATPase) (Palmgren 2001; Zeng et al. 2024). MP is closely related to several key processes, such as hormone biosynthesis (Farmer et al. 2020), nitrate uptake (Fan et al. 2016; Feng et al. 2020), and stomatal operation (Santelia and Lawson 2016; Jezek and Blatt 2017). The changes in MP, either becoming more negative (hyperpolarization) or more positive (depolarization), will affect the activity of numerous voltage-gated ion channels, therefore modulating ion transport across the membrane (Hedrich and Geiger 2017).

When facing fluctuating environments, changes in MP operate upstream of plant adaptive responses. In guard cells (GC), environmental cues induce membrane hyperpolarization for stomatal opening (light and low CO_2_) and membrane depolarization for stomatal closure (abscisic acid, high CO_2_, and high temperature) (e.g. Assmann et al. 1985; Matthews et al. 2020). In roots, oxygen-deprived soil lead to membrane depolarization, resulting in K^+^ leaking via GORK channels and subsequent programmed cell death (Shabala and Pottosin 2014). It has been shown that the ability of sustaining negative MP is essential to confer salt and hypoxia tolerance in plants (Bose et al. 2014; Zeng et al. 2014; Gill et al. 2017). This opens a prospect of detecting MP changes as a proxy to screen and identify tolerant germplasms in breeding programs. However, the conventional method to detect MP changes is the impalement of the membrane with a microelectrode, which is highly time consuming and skill demanding and is prone to various possible artifacts. The method also requires specialized electrophysiological instruments that are not available in breeders’ laboratories. Another method using MP fluorescent probes has been increasingly used in plant research, as it allows for non-invasive and rapid detection of MP changes (Konrad and Hedrich 2008; Cho et al. 2012; Serre et al. 2021). One of them is DISBAC_2_(3)—a probe used in this study—that represents an oxonol dye, a charged and lipophilic molecule that moves between the extracellular solution and the cytosol until the electrochemical equilibrium is reached. When the membrane is depolarized, the dye moves into the cytosol through passive diffusion and binds the lipid membrane (Plášek and Sigler 1996; Wolff et al. 2003). When exposed to excitable light, the dyed samples emit green fluorescence, and the fluorescent light intensifies as the dye accumulates in the cytosol. Conversely, when the membrane is hyperpolarized, less amount of the dye gets into the samples. It was found that this type of slow-moving oxonol-based dye does not accumulate in mitochondria (e.g. Plášek and Sigler 1996).

Here, we present a high-throughput screening approach that allows rapid determination of MP changes in plants by using the fluorescent probe DISBAC_2_(3). By using 3 case studies, we benchmarked this method against conventional microelectrode impalements and demonstrated the feasibility of using this dye for a high-throughput MP screening from various cell types of different species in response to different abiotic stimuli.

## Materials and methods

### Plant materials and conditions

*T. virginiana* plants were purchased from a local nursery shop and grown in a glasshouse on a 14-h-light (62% humidity—26 °C)/10-h-dark (70% humidity—23 °C) cycle at the University of Western Australia. For the dye calibration, 1 pot of *T. virginiana* plants was transplanted to a hydroponic set-up and grown in a growth chamber. Roots were excised and immobilized in the basic salt medium (BSM) solution (0.5 mm KCl, 0.1 mm CaCl_2_) containing NaCl before measuring the MP using MIFE (Shabala et al. 2006) electronics or using DISBAC_2_(3) dye (Cat. No. 44977, Sigma-Aldrich).

Arabidopsis (*A. thaliana* cv. Col-0), barley (*Hordeum vulgare* L cv. Franklin, Naso Nijo and TX9425), rice (*O. sativa* L cv. Nipponbare), pea (*P. sativum* cv. Greenfeast), and corn (*Z. mays* cv. Kelvedon Glory F1) plants were grown from seeds. Except for Arabidopsis, the latter were surface sterilized with 10% commercial bleach for 10 min and then washed with distilled water for 30 min. The paper roll method was used for plant growth. Briefly, sterilized seeds were grown vertically in wet paper in BSM solution, at 24 °C in an incubator, until the roots reached 5 to 8 cm. Arabidopsis seeds were washed with 70% ethanol for 30 s to remove wax, and then surface sterilized with 1% NaClO for 10 min. They were then washed with distilled water for 7 times before being sowed on 1/2 Murashige and Skoog medium plate (1% sucrose, 0.5% phytagel, pH 5.8). After being kept at 4 °C for vernalization for 2 d, the plates were moved into a growth chamber on a 16-h-light/10-h-dark cycle, at 21 °C, for 7 d. Roots were then transferred to BSM solutions containing the treatment: for salinity treatment, 80 mm NaCl was added to BSM for 24 h; for hypoxia, 0.2% agar was added to BSM and the solution was aerated with high-purity N_2_ gas for 1 h (Zeng et al. 2014), and the roots were left in the hypoxia solution for 48 h.

The abaxial epidermis was peeled from a young, green leaf of *T. virginiana* and immersed in the stomatal opening solution (10 mm KCl, 0.05 mm CaCl_2_, 5 mm sucrose, and pH 5.8 to 6) containing 20 mm NaCl. For the stomatal aperture assays and H^+^ flux measurements, a *T. virginiana* plant was dark-adapted overnight before collecting the abaxial epidermal peels from young leaves. After floating on the opening solution for 30 min in the dark, the peels were then immobilized on a glass slide and immersed in the fresh opening solution for 60 min in the dark, and for different time lengths under WL (50 *µ*mol m^−2^ s^−1^), as specified in the Results section.

### MP reading

For membrane impalement, the MP of control and salt-pre-treated *A. thaliana*, *O. sativa*, *T. virginiana*, and *Z. mays* roots was measured using the MIFE amplifier by impaling the roots with 1 m KCl-filled Ag/AgCl microelectrodes (tip diameter of 0.5 *µ*m). The electric gradient across the plasma membrane (MP value) was recorded for 5 to 10 s. For each treatment, at least 20 cells were measured from 4 roots. for DISBAC_2_(3) experiments, the epidermal peels and root samples were dyed either in stomatal opening or BSM solutions containing 2 µm DISBAC_2_(3), for 10 min in the dark, then rinsed with distilled H_2_O and observed under a fluorescent microscope (Leica Microsystems GmbH, Germany) equipped with a light-emitting diode (LED) source (CoolLED pE-300 white), at an excitation wavelength of 450 to 495 nm. Pictures were taken with a camera (DFC295, Leica) mounted on the microscope.

### Image analyses

Stomatal aperture (width of the stomatal pore) and fluorescent intensity were analyzed by the software ImageJ (Schneider et al. 2012). The background of the images of roots was deleted and the green fluorescence intensity (emission wavelength of 500 to 550 nm) of roots was measured specifically by splitting channels in ImageJ and only keeping the green channel. The epidermal region near the GCs and of similar size to the area of interest was selected as background fluorescence to subtract from the GCs fluorescence intensity, hence measuring the accurate fluorescence. For GCs, the green fluorescence was also specifically measured by splitting the channels in ImageJ and measuring only the green channels.

### Ion flux measurements

Net H^+^ fluxes from GCs were measured using the MIFE technique using glass microelectrodes filled with H^+^ ionophore cocktail (Cat. No. 95297, Sigma-Aldrich) as previously described (Shabala et al. 2006). The sign convention was “influx positive.”

### Statistical analysis

SPSS software (version 29.0, IBM, USA) was used for statistical analysis.

## Results

### Comparison between DISBAC_2_(3) fluorescent probe imaging with conventional microelectrode impalement for MP determination

**Figure 1.**
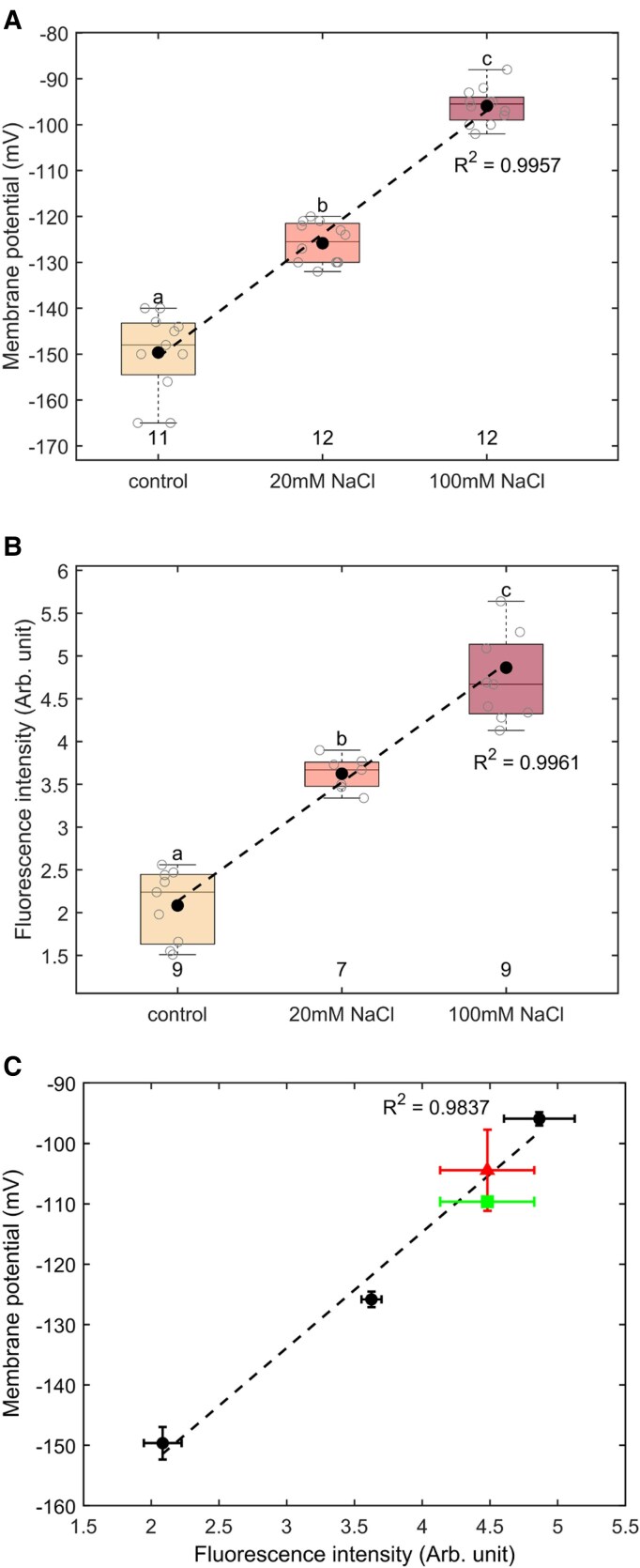
Calibration of fluorescent dye DISBAC_2_(3) using MP measurements on roots. **A)** MP was measured from *T. virginiana* roots with the impalement method under different salt conditions (0, 20, and 100 mm NaCl). **B)** Fluorescence intensity in arbitrary units (Arb. units) of DISBAC_2_(3)-dyed *T. virginiana* roots under the same NaCl conditions as **(A)**. **C)** Correlation between DISBAC_2_(3) fluorescence intensity and MP measurements based on the black points, which are data extracted from **(A)** and **(B)** for 0/20/100 mm NaCl treatment. The green square point indicates the measured MP (with impalement method) and fluorescence intensity (with dye method) from *T. virginiana* roots under 50 mm NaCl. The red triangle point indicates the estimated MP value calculated based on the correlation and measured fluorescence intensity from *T. virginiana* roots under 50 mm NaCl. Mean values ± SE (*n* = 7 to 12) are shown. The correlation dashed line and associated *R*^2^ value are shown in black. Different lowercase values in panels **(A)** and **(B)** indicate a significant difference with *P* < 0.05 (1-way ANOVA, Tukey's Honestly Significant Difference (HSD) test). In the boxplots of **(A)** and **(B)**, the center line represents the median, the box limits indicate the upper and lower quartiles, the whiskers extend to the most distant data points, and the gray circles represent data points.

As the first step, we compared the DISBAC_2_(3) dye with the electrode impalement method for detecting NaCl-induced MP changes. As shown in Fig. 1A, the MP of *Tradescantia virginiana* root, measured by the impalement method, was depolarized after being treated with NaCl and demonstrated a clear NaCl dose-dependent trend (*R*^2^ = 0.995). For the same treatments (0/20/100 mm NaCl), the fluorescence intensity changes of DISBAC_2_(3)-dyed samples were also NaCl dose-dependent (*R*^2^ = 0.996, Fig. 1B), indicating that the fluorescence intensity of DISBAC_2_(3) is positively correlated with membrane depolarization. When the MP measured by impalement was plotted against the fluorescence intensity of DISBAC_2_(3) for the same NaCl treatments, a strong correlation (*R*^2^ = 0.984) was observed in the MP range from −90 to −150 mV (Fig. 1C).

To further verify the accuracy of the dye calibration with the impalement method, we tested the independent data collected from a 50 mm NaCl treatment. There was no significant difference between MP values measured by impalement and values calculated based on the fluorescence intensity and the correlation (Fig. 1C). Thus, it is feasible to use the fluorescence intensity of DISBAC_2_(3)-dyed samples to estimate MP values, after calibrating the dye with the impalement method. This implies that the DISBAC_2_(3) dye method could be used instead of the conventional impalement method to quantify MP values, after appropriate calibration. Additionally, we validated the use of the dye DISBAC_2_(3) by verifying its toxicity and its response to pH and reactive oxygen species (ROS) changes (Supplementary Fig. S1). In this experimental setup, exposure to the dye DISBAC_2_(3) for different durations did not affect root viability of *Pisum sativum* (Supplementary Fig. S1A). Moreover, exposure to different pH and physiologically relevant H_2_O_2_ concentrations did not significantly change the fluorescence intensity of DISBAC_2_(3) (Supplementary Fig. S1B). Taken together, these results show that DISBAC_2_(3) is (i) not toxic in a 30 min window exposure, and (ii) not affected by pH and ROS changes. We next examined 3 case studies to test the efficiency and broadness of this probe in MP determination under different types of treatments.

### Case study 1: Salt stress-induced depolarization in Arabidopsis, barley, rice pea, and corn detected by DISBAC_2_(3)

**Figure 2.**
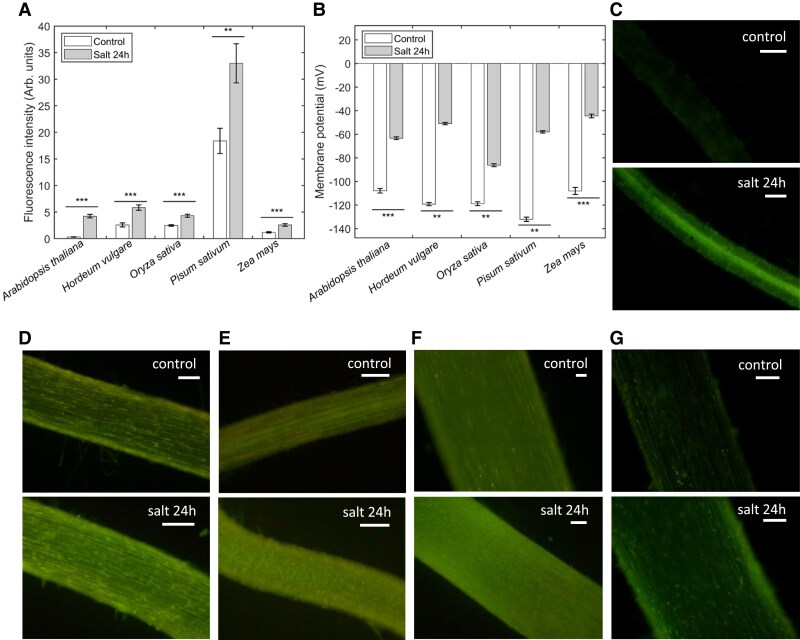
Salt stress-induced depolarization in roots of Arabidopsis, barley, rice, pea, and corn detected by DISBAC_2_(3) dye and the impalement method. **A)** Fluorescence intensity in arbitrary units (Arb. units) measured from DISBAC_2_(3)-dyed roots of Arabidopsis, barley, rice, pea, and corn that were pre-treated with control (BSM) and salt (BSM + 80 mm NaCl) conditions for 24 h. **B)** MP of barley and pea roots was retrieved from the previous study (Bose et al. 2014), and the MP of Arabidopsis, rice, and corn roots was measured by impalement method with MIFE. Mean values ± SE are shown (*n* = 11 to 37 individual roots in panel **(A)**; *n* = 15 to 20 (5 individual roots impaled 3 to 4 times per conditions per species) in panel **(B)**). Asterisks indicate significant differences at **P < 0.01; ***P < 0.001 (Student's *t*-test). **C to G)** For the fluorescence intensity, representative images are shown for every species (*n* = 20 to 22 for *A. thaliana*, *n* = 11 to 14 for *H. vulgare*, *n* = 37 for *O. sativa*, *n* = 22 to 23 for *P. sativum*, and *n* = 17 to 20 for *Z. mays*) taken from control and DISBAC_2_(3)-dyed roots of **(C)**  *A. thaliana*, **(D)**  *H. vulgare*, **(E)**  *O. sativa*, **(F)**  *P. sativum*, and **(G)**  *Z. mays*. The pictures were all corrected with 40% additional brightness and 40% less contrast applied to the pictures to see the fluorescence. Scale bars in white = 100 *µ*m.

For the first case study, we used DISBAC_2_(3) for salt-treated roots of different species to test the broadness of use in various species for MP changes. Changes in fluorescence intensity of DISBAC_2_(3) were compared between control and treatment in Arabidopsis, barley, rice, pea and corn (Fig. 2, A, C to G; Supplementary Fig. S2). These results are consistent with previous results showing the difference in MP in barley and pea (Bose et al. 2014), which suggests that MP changes can be seen through DISBAC_2_(3) intensity changes. The MP of *Arabidopsis thaliana*, *Oryza sativa*, and *Zea mays* was measured with electrode impalement using the microelectrode ion flux estimation (MIFE) system. The difference in mean MP of rice between control and treatment was significantly different, being −119 ± 1.6 and −86 ± 1.2 mV, respectively (Fig. 2B). The difference in mean MP (being of −35 mV for rice) is concordant with the difference in mean fluorescence intensity between control and treatment, the latter being 1.7-fold higher than control (Fig. 2A). This concordance was also found in the other species. For example, the mean fluorescence intensity of treatment in barley is 2.3-fold higher than control, while the MP changes measured previously (Bose et al. 2014) in barley differed by −70 mV (Fig. 2B). Similarly for pea, the mean fluorescence intensity changed by 1.7-fold between control and treatment (treatment being of higher intensity), while the difference in MP measured previously (Bose et al. 2014) is −70 mV (Fig. 2B). It should be noted that the difference in fluorescence intensity between each species for control and treatment is relative to the size and thickness of the roots, unless the confocal scanning microscope is used. This explains somewhat “abnormally high” readings for pea plants under control conditions (Fig. 2A) that are much higher than in other species (which roots are of much smaller diameter). This “apples should be compared with apples” and intensity of fluorescence signal should be calibrated vs MP value for each species.

These results showed that the change in fluorescence intensity of DISBAC_2_(3) dye accurately reflected the change in MP in various species upon salinity exposure, offering a highly convenient tool for breeding programs to screen MP values in stressed plants.

### Case study 2: Hypoxia-induced depolarization in barley root characterized by DISBAC_2_(3)

For the second case study, DISBAC_2_(3) was used to detect the hypoxia-induced depolarization on barley roots to test the applicability of the dye usage in breeding crops for waterlogging stress tolerance. Two barley varieties with contrasting hypoxia tolerance (Naso Nijo—sensitive; TX9425—tolerant; Gill et al. 2017) were used.

**Figure 3.**
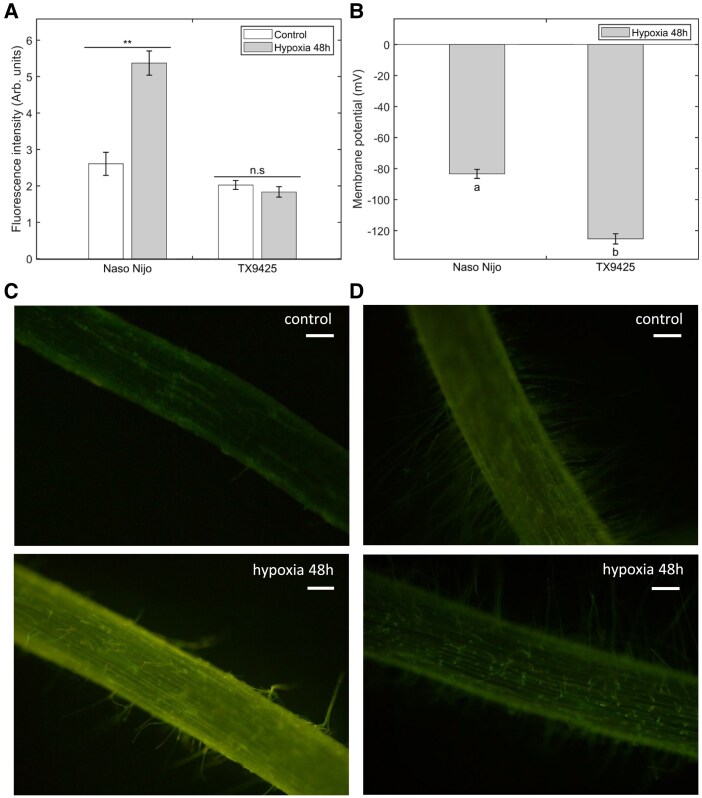
Hypoxia stress-induced depolarization in barley roots detected by DISBAC_2_(3) dye and the impalement method. **A)** Fluorescence intensity in arbitrary units (Arb. units) measured from DISBAC_2_(3)-dyed barley roots of Naso Nijo (sensitive) and TX9425 (tolerant) that were pre-treated with control (aerated BSM) and hypoxia (BSM + 0.2% agar, N_2_ bubbled) conditions for 48 h. **B)** MP of Naso Nijo and TX9425 roots under hypoxia conditions was retrieved from the previous study (Gill et al. 2017). **C** and **D)** Photos of DISBAC_2_(3)-dyed roots of **(C)** Naso Nijo and **(D)** TX9425 under control and treatment conditions. Mean values ± SE (*n* = 25 to 33) are shown. Asterisks indicate significant differences at **P < 0.01, n.s = not significant (Student's *t*-test). Different lowercase values indicate the significant difference at *P* < 0.05 (1-way ANOVA, Tukey's HSD test). The pictures were all corrected with 40% additional brightness and 40% less contrast applied to the pictures to see the fluorescence. Scale bars in white = 100 *µ*m.

The fluorescence intensity of DISBAC_2_(3) was significantly increased in Naso Nijo roots under 48 h of hypoxia stress compared with the control (Fig. 3A), as shown by the pictures (Fig. 3, C and D). In the hypoxia-tolerant variety TX9425, the fluorescence intensity was not significantly different between treatment and control (Fig. 3, A and D). Moreover, the fluorescence intensity of Naso Nijo control, TX9425 control, and TX9425 treatment were not significantly different, indicating the hypoxia-tolerant characteristic of TX9425 (Fig. 3A). These results are consistent with the MP changes measured previously by impalement using MIFE in the same varieties of barley (Fig. 3B), in which a difference of −50 mV in MP can be seen between the tolerant and sensitive varieties (Gill et al. 2017). This difference is observed here in the difference in fluorescence intensity between Naso Nijo and TX9425 in hypoxia stress, being of ∼2.5 Arbitrary units (Arb. units) of fluorescence intensity (Fig. 3A).

These results showed that in addition to salt stress, DISBAC_2_(3) could effectively detect hypoxia-induced depolarization of the plasma membrane and distinct varieties ranging from sensitive to tolerant. Thus, DISBAC_2_(3) could potentially be used for rapid screening of MP changes to identify waterlogging stress-tolerant germplasm.

### Case study 3: Detection of light-induced hyperpolarized MP in GCs

In the third case study, we tested the detection of light-induced hyperpolarization in GC by DISBAC_2_(3). Voltage-sensitive dyes have been used previously on GC to measure membrane depolarization (Konrad and Hedrich 2008; Cho et al. 2012), but they exhibit reduced sensitivity (hence, poor resolution) to the deep hyperpolarized state (Wolff et al. 2003; Serre et al. 2021). To remediate this issue, the initial guard cell MP was slightly depolarized by salt pretreatment at different time points of light exposure. By doing so, the dye can efficiently detect the MP changes in response to light, and the semi-transient measurements allow depiction of these changes over time. Given the strong correlation found between fluorescence intensity and MP changes in *T. virginiana* roots (Fig. 1C), DISBAC_2_(3) could detect the MP changes under 20 mm NaCl (Fig. 1, A and B), which was applied to GC to measure the MP changes in response to light.

**Figure 4.**
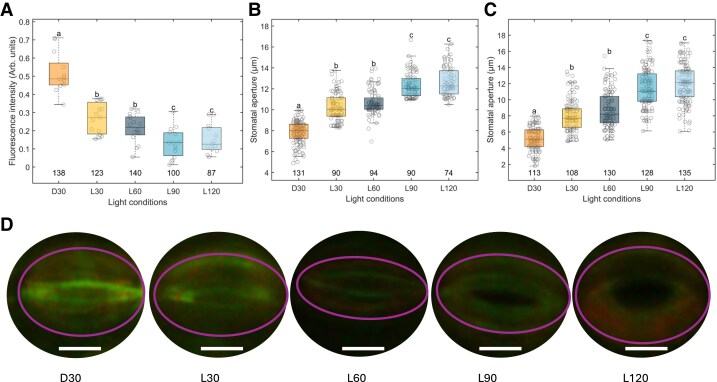
MP of *T. virginiana* GCs measured with DISBAC_2_(3) and stomatal aperture changes. **A)** Fluorescence intensity in arbitrary units (Arb. units) of DISBAC_2_(3)-dyed *T. virginiana* GC pretreated with 20 mm NaCl under different light conditions (D30 = 30 min darkness, L30 = 30 min WL, L60 = 60 min WL, L90 = 90 min WL, and L120 = 120 min WL). The pre-treatment was applied to depolarize the MP, so that the hyperpolarizing effect of light on GC membrane could be detected with the dye. **B)** Stomatal aperture changes in *T. virginiana* GCs without salt treatment under the same light conditions as **(A)**. **C)** Stomatal aperture changes in *T. virginiana* GCs pre-treated with 20 mm NaCl under the same light conditions as **(A)**. The numbers above the *x*-axis represent the replicates of individual GCs measured. Different lowercase values indicate the significant difference at *P* < 0.05 (1-way ANOVA, Tukey's HSD test). In the boxplots of **(A)** to **(C)**, the center line represents the median, the box limits indicate the upper and lower quartiles, the whiskers extend to the most distant data points, and the gray circles represent data points. **D)** Pictures of DISBAC_2_(3)-dyed *T. virginiana* GCs under each light conditions. The pictures here were all corrected with an additional 40% brightness and 20% contrast applied to the pictures to see the fluorescence. The purple ovals circle the GCs. Scale bars in white = 20 *µ*m.

The depolarized state of the GC membrane was seen under dark (Fig. 4, A and D), while under white light (WL), the hyperpolarization of the GC membrane was detected from 30 min under light and continued to be further hyperpolarized until 1.5 h under light (Fig. 4, A and D). While the correlation was approximately linear from dark to 60 min under light (L60), no differences could be seen between 90 min (L90) and 2 h (L120) under light (Fig. 4A). This could be due to either the dye's weaker sensitivity to the hyperpolarized membrane status (Serre et al. 2021), or that there was stable stomatal aperture after 90 min under light, thus likely stable MP between L90 and L120. The stomatal aperture of untreated and treated GC increased over time under light, and aperture changes (Fig. 4, B and C) showed a similar trend with hyperpolarization status (Fig. 4A) under the same light conditions. Hence, it indicated that the similar fluorescence intensity of GC for L90 and L120 (Fig. 4A) was due to no further hyperpolarization in GC as the stomatal aperture remained stable (Fig. 4B). Overall, these results showed that the MP of GCs was significantly hyperpolarized after exposure to light, which is associated with stomatal opening process.

**Figure 5.**
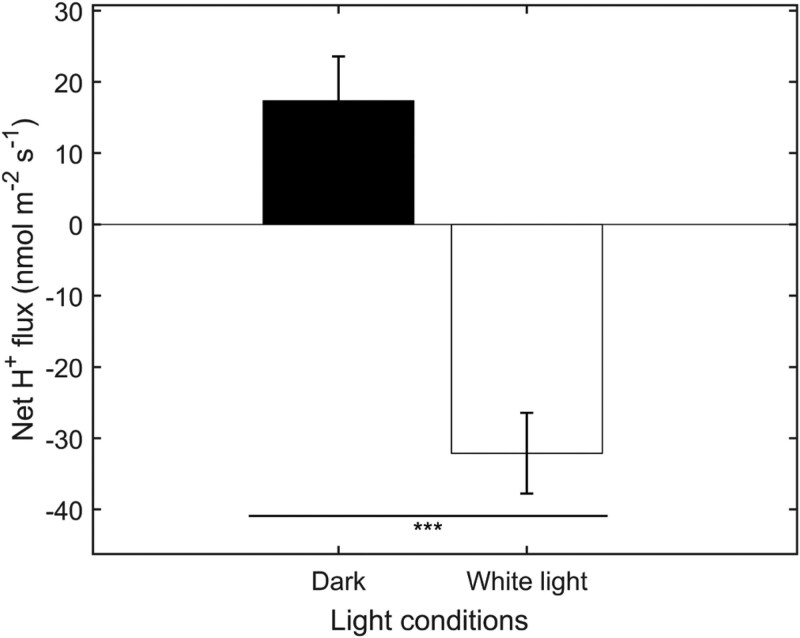
Light-induced proton flux in *T. virginiana* GCs without salt treatment. Net H^+^ fluxes measured under dark and WL for 60 min. Negative value represents H^+^ efflux, while positive values indicate H^+^ influx. Mean values ± SE (*n* = 8 to 12) are shown. Asterisks indicate significant differences at ***P < 0.001 (Student's *t*-test).

To further validate the results of DISBAC_2_(3) for MP hyperpolarization showed in Fig. 4, we tested the changes in proton flux from untreated GC after exposure to 60 min of WL. Stomatal aperture and proton flux changes are good indicators of membrane hyperpolarization, as the plasma membrane H^+^-ATPase is involved in light-induced hyperpolarization in GCs and stomatal opening (e.g. Assmann et al. 1985; Ando and Kinoshita 2018; Matthews et al. 2020). Figure 5 shows H^+^ ions being extruded from GC after exposure to WL for 60 min. These results are consistent with previous experiments (patch-clamp and H^+^ pumping measurements) showing that the H^+^-ATPase extrudes H^+^ ions when phosphorylated, and that light induces phosphorylation of the H^+^-ATPase (Assmann and Schwartz 1992; Roelfsema et al. 2001; Shimazaki et al. 2007). These results therefore suggest that DISBAC_2_(3) effectively detected MP hyperpolarization with an initial depolarization and can be used for rapid screening purposes.

## Discussion

MP is a critical parameter that drives uptake and translocation of all essential nutrients and regulates cell ionic homeostasis, thus conferring adaptation to hostile environmental conditions. However, using conventional methods for MP by impaled microelectrodes requires highly specialized equipment and skills, thus severely limiting its use for phenotyping purposes. In the present study, we demonstrated that the fluorescent dye DISBAC_2_(3) is sensitive enough to detect MP depolarizations, and that this sensitivity is correlated with the conventional impalement method. Through different case studies, we also showed that the dye was able to detect MP changes in various species and different cell types. In particular, the MP depolarization of NaCl-treated roots of 6 species (*T. virginiana*, Arabidopsis, barley, rice, pea, and corn) was accurately reflected in the changes in fluorescence intensity of DISBAC_2_(3)-dyed samples (Figs. 1 and 2; Supplementary Fig. S2). Similarly, the dye method is applicable to detect MP depolarization induced by other forms of stress, including hypoxia (Fig. 3). These findings show the broadness of use of DISBAC_2_(3) to detect MP depolarization induced by different stresses in various species. Given its applicability, DISBAC_2_(3) could be used for rapid screening of plant populations of interest (e.g. double haploid lines to identify quantitative trait loci for MP maintenance).

In the third case study, we used the dye to detect hyperpolarization in GCs. A hyperpolarized membrane status prevents the dye from interacting with the membrane (Wolff et al. 2003). Hence, caution should be taken when measuring hyperpolarized values. With pre-induced depolarization, hyperpolarized values were found here in GC dyed with DISBAC_2_(3), although the fluorescence intensity was smaller than depolarized values. By verifying with the stomatal aperture and GC steady-state H^+^ ion flux measured under the same light conditions, our results (Figs. 4 and 5) demonstrated the feasibility of using DISBAC_2_(3) to detect hyperpolarized membrane. GC movements, coordinated by the MP, have major impact on plant photosynthetic performance, and transpiration via open stomata plays a crucial role in crop production and adaptability in drier environments (Lawson and Blatt 2014; Jezek and Blatt 2017). Rapidly and effectively screening for optimal, dynamic GC responses to environmental cues, via measuring MP changes, may become a solution to identify crops with high adaptive potential.

In this study, we showed that DISBAC_2_(3) is sensitive to MP changes within the range of −90 to −150 mV. However, as DISBAC_2_(3) shows lower resolution to negative values, its detection limitation remains to be checked, for instance by examining the fluorescence intensity changes under voltage clamp conditions. In addition, calibrating the dye in target species with the conventional impalement method allows accurate estimation of the MP values with the dye in the targeted species (Fig. 1C). This correlation between the DISBAC_2_(3) dye and conventional impalement methods remain species-specific, thus impaling the membrane of targeted species is required to have accurate estimation, as from one species to another, the fluorescent intensity of the dye may vary. The dye Arb. units can then be used as a proxy to MP values, hence making the dye a useful tool to identify qualitative and quantitative MP changes.

In conclusion, this study demonstrates the utility for DISBAC_2_(3) for rapidly detecting MP changes (qualitatively and quantitatively), revealing potential for broader use in plant field research, especially for high-throughput screening for varieties tolerant to abiotic or biotic stresses. This protocol may be considered “high-throughput” compared with the conventional method because the number of samples tested and analyzed per day can be exponentially increased using automated (robotic) systems for specimens handling and analysis. In the conventional way, high-skilled researchers can test from 20 to 30 samples per day, while the automated way, relying on taking images and analyzing the fluorescence intensity, could test and analyze many hundreds of samples daily when combined with machine learning. Abiotic stress is estimated to cost around US$80 billion per year in drought-affected crop losses, and around US$74 billion per year in losses from waterlogging damage (Kaur et al. 2020; Razzaq et al. 2021). There is an urgent need to “speed-up” the identification of stress-tolerant germplasm. Regaining abiotic stress tolerance lost during domestication (Palmgren and Shabala 2024) combined with rapid screening offered by voltage-sensitive dyes may become a solution for breeders to resolve these economic losses.

## Supplementary Material

kiaf560_Supplementary_Data

## Data Availability

Data available on request.

## Supplementary data

The following materials are available in the online version of this article.

**Supplementary Figure S1**. Toxicity of DISBAC_2_(3) and effects of non-voltage factors on DISBAC_2_(3) signal.

**Supplementary Figure S2**. Salt stress-induced depolarization in the roots of Arabidopsis, barley, rice, pea, and corn detected by DISBAC_2_(3) dye and the impalement method.

## Dive Curated Terms

The following phenotypic, genotypic, and functional terms are of significance to the work described in this paper:

GORK Gramene: AT5G37500
GORK Araport: AT5G37500
