# Supplementary material for: The fluorescent probe DISBAC2(3) provides a high-throughput screening tool for evaluating abiotic stress tolerance in plants
Source: Plant Physiol. 2025 Nov 4;199(3):kiaf560. doi: 10.1093/plphys/kiaf560 (PMC12622375; doi:10.1093/plphys/kiaf560)
Supplement: kiaf560_Supplementary_Data [file kiaf560_supplementary_data.pdf]

A

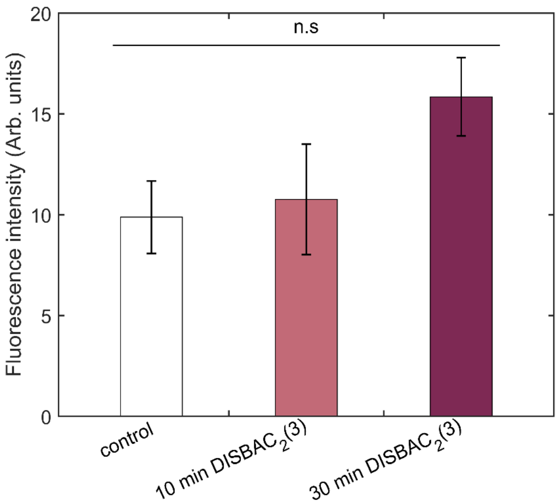

B

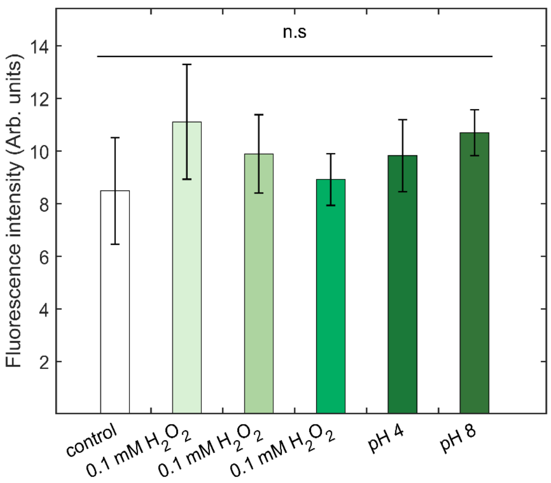

Supplementary Figure S1. Toxicity of DISBAC$_2$(3) and effects of non-voltage factors on DISBAC$_2$(3) signal. **A)** Fluorescence intensity in arbitrary units (Arb. units) of propidium iodide (PI) in *P. sativum* roots after exposure to DISBAC$_2$(3). The toxicity of the dye DISBAC$_2$(3) on biological samples was measured as the changes in fluorescence intensity of the dye PI. Mean values ± *SE* (*n* = 6 – 8) are shown; n.s = not significant at $P<0.05$ (one-way *ANOVA*, *Tukey's HSD Test*). **B)** Fluorescence intensity of DISBAC$_2$(3) in *P. sativum* roots in different pH and H_2O_2 concentrations. The effect of non-voltage factors (pH and ROS changes) was measured as the difference in fluorescence intensity of DISBAC$_2$(3). Mean values ± *SE* (*n* = 6 – 13) are shown; n.s = not significant at $P<0.05$ (one-way *ANOVA*, *Tukey's HSD Test*).

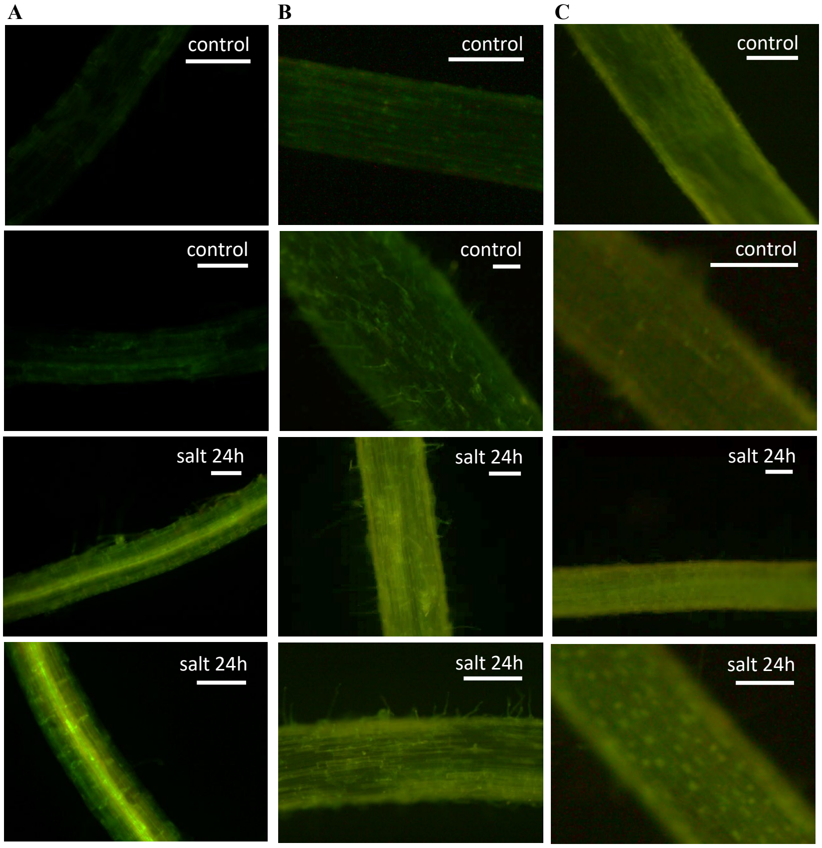

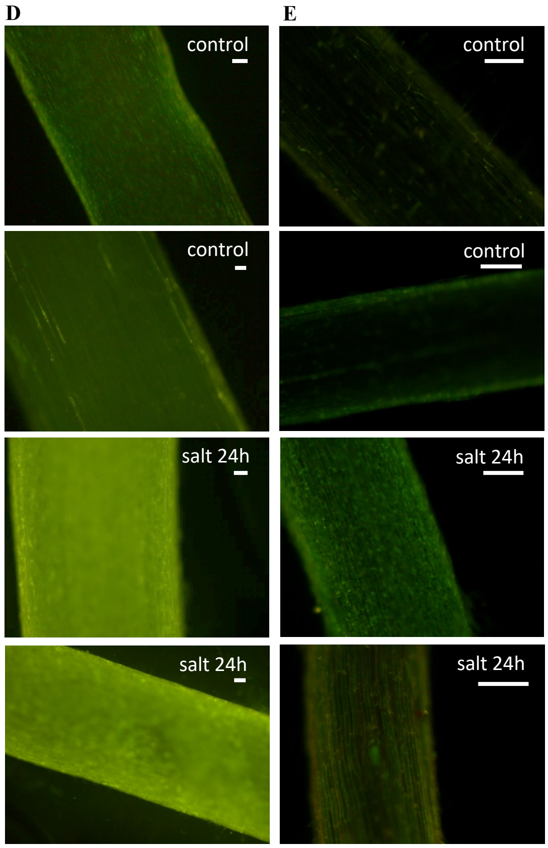

Supplementary Figure S2. Salt stress-induced depolarization in the roots of Arabidopsis, barley, rice, pea and corn detected by DISBAC$_2$(3) dye and the impalement method. **A-E)** For the fluorescence intensity, representative images are shown for every species (n = 20 – 22 for *A. thaliana*, n = 11 – 14 for *H. vulgare*, n = 37 for *O. sativa*, n = 22 – 23 for *P. sativum*, and n = 17 – 20 for *Z. mays*), here are shown other representative pictures of control and treatment DISBAC$_2$(3)-dyed roots of **(A)** *A. thaliana*, **(B)** *H. vulgare*, **(C)** *O. sativa*, **(D)** *P. sativum*, and **(E)** *Z. mays*. The pictures were all corrected with 40 % additional brightness and 40 % less contrast applied to the pictures to see the fluorescence. Scale bars in white = 100 µm.
